# The effects of add-on corticosteroids on renal outcomes in patients with biopsy proven HIV associated nephropathy: a single centre study from South Africa

**DOI:** 10.1186/s12882-019-1208-2

**Published:** 2019-02-06

**Authors:** Nicola Wearne, Charles R. Swanepoel, Maureen S. Duffield, Bianca J. Davidson, Kathryn Manning, Nicki Tiffin, Andrew Boulle, Brian L. Rayner, Priyanka Naidu, Ikechi G. Okpechi

**Affiliations:** 1 Division of Nephrology and Hypertension, Groote Schuur Hospital, University of Cape Town, Cape Town, South Africa; 2 Kidney and Hypertension Research Unit, University of Cape Town, Cape Town, South Africa; 3 Division of Pathology, Department of Clinical Laboratory Services, University of Cape Town, Cape Town, South Africa; 4 Department of Medicine, Statistical Analyst, University of Cape Town, Cape Town, South Africa; 5 CIDER: School of Public Health and Family Medicine, University of Cape Town, Cape Town, South Africa; 6 Department of Undergraduate Medicine, University of Cape Town, Cape Town, South Africa

**Keywords:** HIV, HIV associated nephropathy, Corticosteroids

## Abstract

**Background:**

The aim of this study was to assess, the efficacy and safety of add-on corticosteroids to antiretroviral therapy [ART] in patients with biopsy proven HIV associated nephropathy.

**Methods:**

All included patients had histological evidence of either collapsing or non-collapsing focal segmental glomerulosclerosis (FSGS) or podocyte and/or parietal cell hypertrophy or hyperplasia. All patients had evidence of tubulointerstitial inflammation with microcysts. Patients were randomized to ART with the addition of 1 mg/kg of corticosteroids [ART+C] or remained in the group [ART Alone] and followed for 2 years. A repeat biopsy was performed at 6 months.

**Results:**

Twenty-one patients were randomized to [ART+C] and 17 to [ART Alone]. The baseline estimated glomerular filtration rate (eGFR) was significantly lower in the [ART+C] vs. [ART Alone] group [35mls/min/1.73m^2^ vs. 47 mls/min/1.73m^2^, *p* = 0.015]. The [ART+C] cohort had a statistically significant improvement in median (eGFR) from baseline to last follow up compared with [ART Alone] i.e. [Δ = 25mls/min (IQR: 15;51) vs 9 mls/min (IQR: 0–24), *p* = 0.008].

There were no statistically significant differences between the groups when proteinuria and histology were analyzed. There were 8 deaths during the trial period, 7 from [ART+C] (Log rank *p* = 0.071).

**Conclusions:**

In the [ART+C] cohort there was a significant improvement in eGFR over 2-years with increased mortality. Routine corticosteroid use cannot currently be recommended. Further investigation to define which subgroup of this cohort would safely benefit from the positive effects is required.

**Trial registration:**

ISRCTN study ID (56112439] was retrospectively registered on the 5 September 2018.

**Electronic supplementary material:**

The online version of this article (10.1186/s12882-019-1208-2) contains supplementary material, which is available to authorized users.

## Background

Despite the era of antiretroviral therapy [ART], human immunodeficiency virus [HIV]–related kidney disease still carries a significant risk of end-stage renal disease (ESRD) [1]. From available data in Africa the prevalence of chronic kidney disease (CKD) in HIV-infected patients is reported to be 38% in Nigeria [2], 33.5% in Zambia [3], 20% in Uganda [4], 11.5% in Kenya [5], and 5.5–6% in South Africa [6, 7]. The regional variations in CKD prevalence is likely due to differences in reporting methods, sampling, definition of CKD, access to health care and genetic factors [8].

The recent [Kidney Disease Improving Global Outcomes] KDIGO Controversies Conference publication, endorsed a pathological classification of HIV-related kidney diseases. The classification encompassed glomerular-, tubulointerstitial- and vascular-dominant lesions as well as other lesions seen in the setting of HIV [9]. Classic HIV associated nephropathy [HIVAN] is defined as a collapsing glomerulopathy with interstitial inflammation, microcysts and tubular injury [9]. The interstitial component includes a diffuse inflammatory cell infiltrate which may include plasma cells [8, 10–13]. Numerous studies describe the release of several inflammatory mediators e.g. interleukin 6, nuclear factor kappa B [14] and transforming growth factor β [15]. These mediators promote interstitial inflammation and subsequent fibrosis [14, 16, 17].

Mortality is reduced when initiating antiretroviral therapy [ART] in patients with HIVAN [11, 18]. However despite ART improving mortality and renal impairment there are still a subgroup of patients where CKD progresses regardless of ART [11]. This may be explained by the kidneys acting as a viral reservoir for HIV nucleic acid, even on ART [19–21].

A number of old studies have evaluated the efficacy and safety of corticosteroids in the treatment of classic HIVAN [22–24]. However, these studies were limited by their retrospective, non-randomized design together with different ART and steroid regimens. There has also been safety concerns due to increased risk of infections [18]. To the best of our knowledge only one retrospective study of 19 patients has assessed the use of corticosteroids for the treatment of HIVAN in the triple therapy era of ART and demonstrated a significant improvement in estimated glomerular filtration rate [eGFR] in the corticosteroid arm [24]. Further trials to assess corticosteroid benefit have therefore been recommended [18, 21].

The aim of our study was to assess the balance between improvement in eGFR, proteinuria and adverse events with adjuvant corticosteroids to ART in patients with HIVAN. Our hypothesis was that there would be a greater improvement in eGFR in the corticosteroid cohort due to the dampening effect of inflammation in the interstitial compartment in those patients with HIVAN. The importance is underscored by the high prevalence of CKD in HIV positive patients in Africa and the lack of prospective treatment studies.

## Methods

This was a prospective, open labelled trial to investigate the response to corticosteroids as adjuvant treatment to ART in patients with glomerular and interstitial features of HIVAN. The study was approved by the Human Research Ethics Committee of the University of Cape Town [HREC:241/2007]. The trial was registered with the ISRCTN registry [ISRCTN study ID (56112439].

### Study population

HIV positive patients (defined by a positive Elisa test) with unexplained renal impairment, proteinuria and/or haematuria underwent renal biopsy as per best clinical practice for the period [April 2010–May 2015]. Inclusion criteria for the study included all ART naïve patients with histologically proven HIVAN. This was defined as 1) collapsing glomerulopathy 2) focal segmental glomerulosclerosis or 3) podocyte hypertrophy and/or hyperplasia plus all biopsies required the presence of chronic tubulointerstitial inflammation with plasma cells, lymphocytes and microcysts. Further inclusion criteria included age > 18 years, written informed consent, no prior ART exposure (ART naïve) or newly initiated on ART for less than 2 weeks prior to renal biopsy; and initiation of ART within one-month post biopsy. Exclusion criteria included any active infection (including Kaposi sarcoma and active cytomegalovirus), eGFR of < 10 ml/min/1.73m^2^ and an inability to follow up at our centre.

### Study outcomes

The primary outcome was to assess, in patients with HIVAN, the change in eGFR and proteinuria between the [ART+C] and [ART Alone] cohorts from baseline to 24 months. Secondary outcomes were to monitor for adverse events and to review whether there was improvement in histology; specifically podocytopathy, interstitial fibrosis, lymphocytic and plasma cell infiltration from baseline. In consented patients, comparisons were made with a repeat renal biopsy at 6 months.

### Treatment regimen

We allocated consecutive patients to ART plus corticosteroids [ART + C] or [ART Alone]. Consecutive patients were assigned their treatment by randomly selecting a sealed opaque envelope with the treatment allocation. Both investigator and patients were not blinded during the study period as additional prophylactic treatment was required to prevent opportunistic infections in those receiving [ART+C]. The ART regimen used in the trial was determined by the Department of Health policy on ART roll-out during the study period.

Patients in the [ART+C] arm were commenced on 1 mg/kg of prednisone within 2 weeks of starting ART (maximum dose was 60 mg/day irrespective of body weight). Where patients had tuberculosis [TB] and were on rifampicin at the start of the study, prednisone dose was increased to 1.5 mg/kg with a maximum dose of 90 mg/day. Prednisone was tapered over a 6-month period by 10 mg per month. Treatment with angiotensin converting enzyme inhibition (ACEi) or angiotensin receptor blockade (ARB) was also commenced in those patients whose BP would allow and where hyperkalaemia was not a complication. Isoniazid and cotrimoxazole was started in the [ART+C] group for TB and pneumocystis jirovecii prophylaxis.

### Baseline measurements

At baseline, demographic features (age, gender, and ethnicity), clinical features including weight, systolic and diastolic blood pressures (SBP and DBP), renal function; serum creatinine (μmol/L), eGFR (mL/min/1.73 m^2^), and urine protein/creatinine ratio (uPCR)(gm/mmol), CD4 count (cells/mm^3^),HIV viral load (log10cps/ml), haemoglobin (Hb)(g/dL) and cholesterol (mmol/L) were obtained and documented. In keeping with our centre’s protocol for assessing patients with glomerular pathologies, various serological markers were also assessed and included anti-nuclear antibody, anti-double-stranded DNA, RPR, hepatitis B and C, anti-streptolysin titre, anti-DNase B titre and total complement assays (C3 and C4) to exclude other forms of kidney disease. All variables were obtained within 2 weeks of the renal biopsy. The eGFR was calculated according to the *Chronic Kidney Disease* Epidemiology Collaboration [*CKD*-*EPI*] eq. [25]

### Follow-up visits

Patients were followed up monthly for 6 months and then every 3 months for 2 years. At each visit the following assessments were performed: blood pressure (BP), serum creatinine, eGFR, Hb and uPCR. Serum cholesterol, viral load and CD4 counts were measured every 3 months. Mortality data was obtained from death certificates, patient folders, records from HIV clinics and family members.

### Histology

An initial renal biopsy was performed in all participating patients and the obtained specimen was processed for light microscopy with haematoxylin and eosin (H&E), methenamine silver and periodic acid-Schiff (PAS) stains. Standard techniques were performed to demonstrate IgA, IgM, IgG and C3 for immunohistochemistry and electron microscopy. A single histopathologist [MD] and nephrologist [NW] reviewed all biopsies. The biopsies were reviewed for glomerular or tubulointerstitial features of HIVAN as described in the inclusion criteria.

The degree of interstitial fibrosis, lymphocytic infiltrate in the interstitium and amount of microcysts present in the interstitium were graded from 0 to 4 according to the following grading system [0 < 5%; 1 = 5–25%; 2 = 26–50%; 3 = 51–75%; 4 > 75%.] The percentage of plasma cells within the lymphocytic infiltrate was graded 0–4 [0 = 0; 1 < 5%; 2 = 6–15%; 3 = 16–30% and 4 > 30%.] An upper limit of the plasmacytic infiltrate was 30%, as beyond this the infiltrate may have been considered part of a lymphoproliferative disorder. The presence or absence of podocyte and parietal cell hypertrophy and hyperplasia was determined [0 = none, 1 = present]. In consenting patients, a repeat renal biopsy was performed to assess histological changes at 6 months. An interval change was interpreted as “improving” if there was a reduction in the grading seen at repeat biopsy. The assessment of repeat histology was blinded to the histopathologist [MD] and nephrologist [NW].

### Statistics

Data was analysed using Stata version 14.2 (Stata Corporation, College Station, TX). Missing variables for follow up eGFR and uPCR were imputed using last observation carried forward for all patients who died or were lost to follow up in the trial period. Any patient with less than 3 months follow up data was excluded from the final analysis. Continuous variables were summarised as medians with interquartile range [IQR], while categorical variables were summarised using frequencies and proportions. Continuous variables were compared using the Wilcoxon rank-sum test, and categorical data was compared using Fisher’s exact test.

A LOESS smoothing function was used to describe the relationship between follow-up time on ART and eGFR, stratified by steroid treatment. A smooth curve was generated through the scatter plot using locally weighted regression.

Multivariable linear regression analyses was performed to analyse the association between change in eGFR and uPCR, and treatment between [ART+C] and [ART Alone] when adjusted for known and potential confounders including age, gender and ACEi/ARB treatment.

Fisher’s Exact test used to compare baseline histological features between groups. Histological change from baseline to repeat biopsy was categorised into “improving” versus “no change” or “worsening” according to the original histological grading as described in the methods. Fisher’s exact test was used to compare binary histological change (yes/no) at repeat biopsy between [ART + C] and [ART Alone].

A Kaplan-Meier survival curve was used to illustrate survival probability for [ART + C] and [ART Alone] in all patients. Log-rank test was used to compare survival curves between treatment groups. The results with *p* < 0.05 were interpreted as statistically significant where appropriate.

## Results

**Fig. 1:**
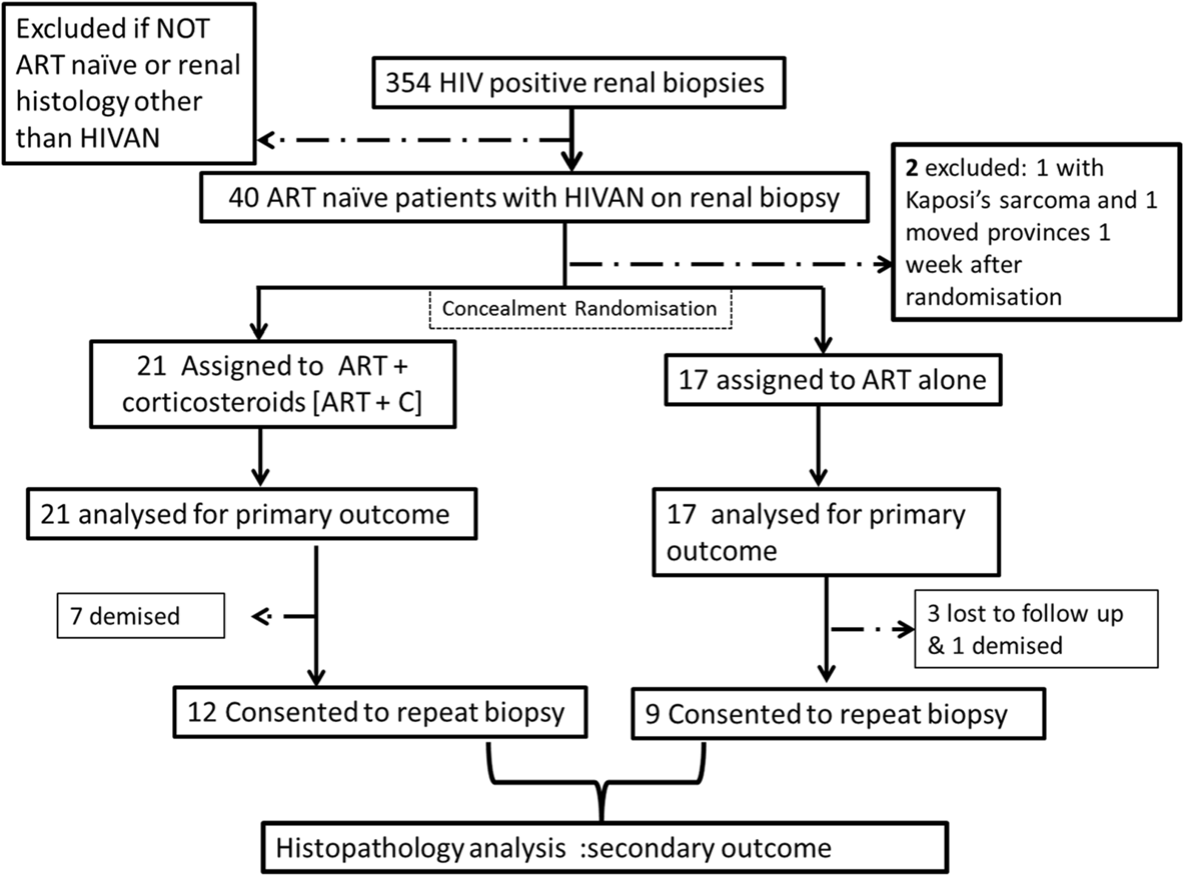
Study flow chart for the trial

A total of 1131 renal biopsies were performed during the trial period. Of these, 354 [31.3%] were from HIV-infected patients (Fig. 1: Study flowsheet for the trial). All patients had a clinical indication for biopsy. There were 38 patients who met criteria for inclusion into the trial; of these, 21 patients (55.3%) were treated with [ART+C] while 17 patients (44.7%) were treated with [ART Alone]. Table 1 describes the baseline characteristics of the cohort.

**Table 1:** Baseline demographic, clinical and biochemical parameters

	ALL *N* = 38	[ART + C] *N* = 21	[ART Alone] *N* = 17	*P* Value*
Demographics				
Gender (female), n[%]	25 [65.8%]	14 [66.7%]	11 [64.7%]	0.899^a^
Age (years) median IQR	33 [2;39]	36 [27;42]	28 [25;34]	0.050^b^
Clinical parameters				
Systolic BP (mmHg) median [IQR]	118 [110;125]	118 [110;125]	118 [110;121]	0.668^b^
Diastolic BP (mmHg median [IQR]	77 [71;83]	76 [65;80]	77 [71;84]	0.561^b^
Diabetes; n [%}	1 [2.6%]	1 [4.8%]	0	1.000^a^
Hypertension, n [%}	7 [18.4%]	5 [23.8%]	2 [11.8%]	0.306^a^
Tuberculosis at baseline, n [%}	10 [26.3]	7 [33.3%]	3 [17.6%]	0.242^a^
Commenced on ACE/ARB; n [%}	27 [71.1%]	14 [66.7%]	12 [70.6%]	0.519^a^
ART regimen at baseline, n [%}				0.332^a^
- D4T/3TC/EFV or NEV	20 [52.6%]	11 [52.4%]	9 [52.9%]	
- ABC/3TC/EFV or NEV	9 [23.7%]	7 [33.3%]	2 [11.8%]	
- ZVD/3TC/EFV or NEV	6 [15.8%]	2 [9.5%]	4 [23.5%]	
- Other/unknown	3 [7.9%]	1 [4.8%]	2 [11.8%]	
Biochemical parameters				
eGFR (ml/min/1.73m^2^) median[IQR]	43.5 [26;70]	35 [18;46]	47 [39;97]	0.015^b^
eGFR (ml/min/1.73m^2^) mean [SD]	50 [32.8]	37.8 [26.0]	65.1 [34.8]	
CD4 (cells/mm^3^) median[IQR]	158 [63;309]	158 [36;272]	173 [104;352]	0.437^b^
HIV VL (Log 10) (cps/ml) median[IQR]	4.77 [3.87;5.29]	4.77 [4.5;5.29]	4.69 [2.75;5.3]	0.5623^b^
Albumin (g/L) median[IQR]	30 [25;33]	29 [24;32]	30.5 [27;36]	0.262^b^
uPCR (g/mmol) median[IQR]	0.36 [0.14;0.54]	0.43 [0.17;0.52]	0.23 [0.13;0.60]	0.617^b^
Haemoglobin (g/dL) median[IQR]	8.1 [7.3;10.2]	8.0 [7.4;9.9]	8.1 [7.3;10.2]	0.872^b^
Cholesterol(mmol/l) median[IQR]	4.5 [3.6;5.6]	4.4 [3.3;5.1]	4.8 [3.9;6.0]	0.347^b^

*ART + C* Antiretrovirals + corticosteroids, *ART Alone* Antiretrovirals alone, *IQR* interquartile range, *BP* Blood pressure, *ACEi* angiotensin converting enzyme inhibitor, *ARB* angiotensin receptor blocker, *D4T* Stavudine, *3TC* Lamivudine, *NEV* Nevirapine, *ABC* Abacavir, *EFV* Efavirenz, *ZVD* Zidovudine, *uPCR* urine protein-to-creatinine ratio, *HIV VL* HIV viral load, *eGFR* estimated glomerular filtration rate, *SD* standard deviation. a = Fishers Exact test, b = Wilcoxon Rank-Sum test, * *p*-value comparison of [ART+C] vs [ART Alone]

Overall, there were more females (65.8%), median age was 33 (IQR: 25–39) years, median SBP and DBP was 118 (110–125) mmHg and 77 (71–83) mmHg, respectively. All patients included in the trial were of black African ethnic origin. Only one patient (2.6%) had diabetes while 7 [18.4%] were known with hypertension. There were ten patients (26.3%) who were diagnosed with TB at time of presentation. Patients were initiated on an ACE-I or ARB if their BP could tolerate it and if hyperkalaemia was not a complication. Overall 71.1% of patients were treated with an ACE-I or ARB ([ART +C] 14/21 and [ART Alone] 12/17). All patients received triple regimen ART therapy. The most common initial ART regimen [52.6%] consisted of stavudine (D4T), lamivudine (3TC) and efavirenz (EFV) /nevirapine (NEV). The other ART combinations are detailed in Table 1; no patient received tenofovir.

There were 2 variables that were significantly different between the groups. The first was median eGFR which was lower in the [ART+C] arm [35mls/min/1.73m^2^ vs. 47 mls/min/1.73m^2^, *p* = 0.015]. The second was median age where the [ART Alone] group was younger [28 yrs. vs 36 yrs., *p* = 0.050](Table 1). All serological markers tested to exclude other causes of kidney disease were negative. The baseline histological features are outlined in Table 2. There were no statistically significant differences histologically between the groups.

**Table 2:** Baseline histological features

Histological features	Total (%)	ART + C (%)	ART Alone (%)	*P* Value^a^
	*N* = 38	*N* = 21	*N* = 17	
Classic HIVAN	68.4%	66.7%	70.6%	0.791
FSGS [NOS] with tubulointerstitial inflammation and microcysts	26.3%	33.3%	17.6%	0.268
Podocytopathy with tubulointerstitial inflammation and microcysts	5.3%	0%	11.8%	0.103
Interstitial disease				
Lymphocytic infiltration[grading]				
0	5.3%	4.8%	5.9%	0.799
1	34.2%	8.6%	41.2%	
2	26.3%	23.8%	29.4%	
3	21.1%	28.6%	11.8%	
4	13.2%	14.3%	11.8%	
Plasmacytic infiltration[grading]				
0	5.3%	4.8%	5.9%	0.533
1	28.9%	28.6%	29.4%	
2	18%	9.5%	29.4%	
3	13.2%	19.0%	5.9%	
4	34.2%	38.1%	29.4%	
Interstitial fibrosis [grading]				
0	5.3%	0%	11.8%	0.389
1	28.9%	14.3%	35.3%	
2	15.8%	14.3%	11.8%	
3	36.8%	23.8%	29.4%	
4	13.1%	4.8%	11.8%	
Microcysts [grading]				
0	31.6%	23.8%	41.2%	0.698
1	28.9%	33.3%	23.5%	
2	28.9%	33.3%	23.5%	
3	10.5%	9.5%	11.8%	
4	–	–	–	

*ART + C* Antiretrovirals + corticosteroids, *ART Alone* Antiretrovirals alone, *FSGS* Focal segmental glomerulosclerosis, *NOS* Not otherwise specified

Microcysts, lymphocytic infiltrate and fibrosis in the interstitium was graded 0–4: [0 < 5%; 1 = 5–25%; 2 = 26–50%; 3 = 51–75%;4 > 75%] The percentage of plasma cells within the lymphocytic infiltrate was graded [0 = 0; 1 < 5%; 2 = 6–15%; 3 = 16–30% and 4 > 30%]

Podocytes and parietal cell hypertrophy/hyperplasia were graded 0 = absent; 1 = present

^a^ = the Fisher’s exact test used to compare baseline histological features between groups

### Changes in clinical and biochemical parameters from baseline until last follow–up

**Fig. 2:**
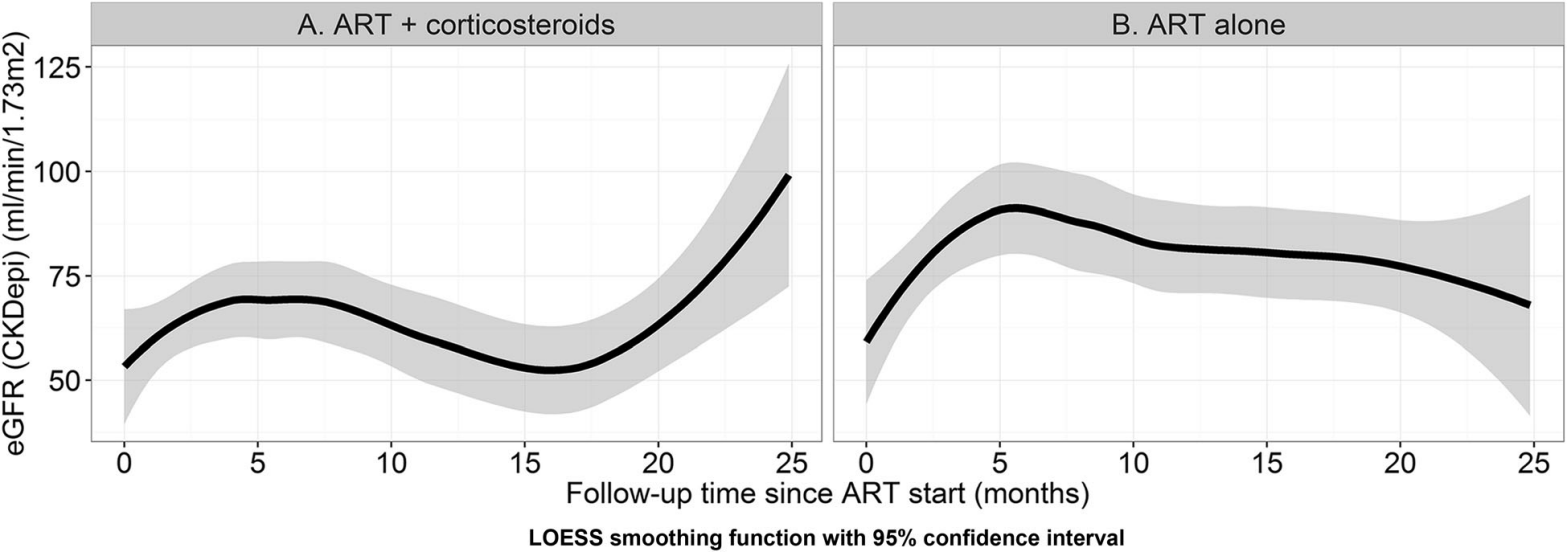
eGFR by duration on ART with and without the addition of corticosteroids. Footnote. LOESS smoothing function with 95% confidence interval

**Fig. 3:**
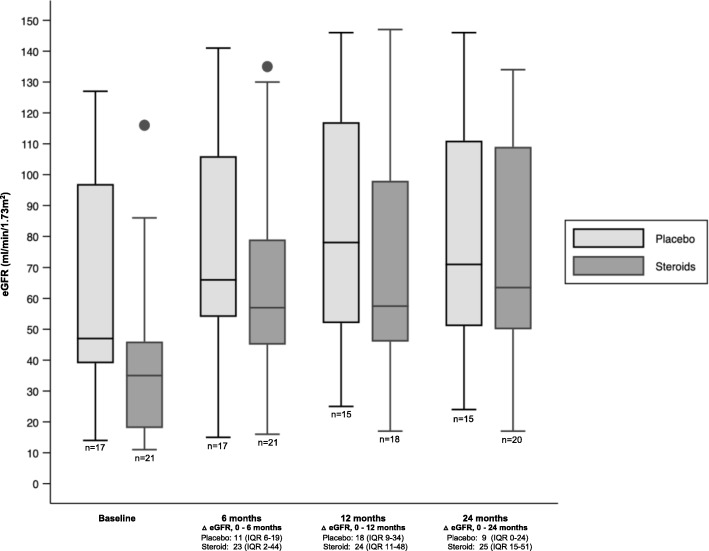
Box and whisker plot representing the median eGFRs with IQRs at 6, 12- and 24-month periods

All patients were analyzed as per intention to treat for the primary outcome. Additional file 1 Table S1 includes all changes in median CD4 and viral load at 3-,6-, 12- and 24-months. Over the study period the eGFR improved in both the groups (Fig. 2: eGFR by duration on ART with and without the addition of corticosteroids.) Figure 3 describes the median eGFR and IQR at 6, 12- and 24-month periods. The group receiving [ART+C] had a statistically significant improvement in median eGFR from baseline to last follow-up compared with [ART Alone] i.e. [Δ = 25mls/min (IQR: 15–51) vs 9 mls/min (IQR: 0–24), *p* = 0.008] (Table 3).

**Table 3:** The change [Δ] in clinical and biochemical parameters at 24 months

Median change [Δ] in parameters at 24 months [IQR]	[ART + C]	[ART Alone]	*P* Value^a^
Clinical parameters			
Systolic BP mmHg [IQR]	10 [−25; 11]	6 [2; 18]	0.466
Diastolic BP mmHg [IQR]	−6 [−20;4]	1 [−5; 7]	0.177
Biochemical parameters			
Change in eGFR (ml/min/1.73m^2^) [IQR]	25 [15;51]	9 [0;24]	0.008
Change in uPCR (gm/mmol) [IQR]	−0.13 [−0.25; −0.08]	−0.12 [−0.55; −0.06]	0.691
Change in Haemoglobin (gm/dL)[IQR]	3.4 [0.3; 4.7]	3.6 [2.1; 4.8]	0.480
Change in Albumin (gm/L)[IQR]	5 [2; 12]	6 [4;15]	0.445
Change in Cholesterol (mmol/l)[IQR]	0.2 [−0.10; 1.7]	0.3 [−1.5; 0.9]	0.577

*ART + C* Antiretrovirals + corticosteroids, *ART Alone* Antiretrovirals alone, *BP* Blood pressure, *eGFR* estimated glomerular filtration rate, *uPCR* protein creatinine ratio, a = Wilcoxon Rank-Sum test

Additional file 2 Table S2 describes all eGFR values at baseline and last follow-up. Proteinuria improved in both groups over the trial period however the change in proteinuria was not statistically significant between the groups. [(ART+C) Δ = − 0.13 g/mmol (− 0.25;-0.08) versus (ART Alone) Δ = − 0.12 g/mmol (− 0.55;0.06) *p* = 0.691] (Table 3). There was no clinically significant change in BP, Hb, cholesterol or albumin over the trial period.

Table 4 demonstrates the multivariable linear regression analysis for change in eGFR and uPCR at 24 months. After adjustment for baseline eGFR, age, ACEi and sex the change in eGFR with the addition of corticosteroids from baseline to 24 months trended towards significance (β coefficient: 18.63 ml/min/1.73 m^2^, 95% CI [− 2.26–39.52]; (*p* = 0.078) (Table 4). There was no association observed for change in uPCR (β coefficient: 0.09 g/mmol, 95% CI -0.19-0.33); (*p* = 0.525) over the study period.

**Table 4:** Multivariable linear regression analysis for Change in eGFR and uPCR at 24 months

	Δ in eGFR (ml/min/1.73m^2^) at 24 months		Δ in UPCR (gm/mmol) (at 24 months	
	β Coefficient (95%CI)	*P* = value	β Coefficient (95% CI)	*P* = value
On steroids	18.63 [−2.26–39.52]	0.078	0.09 [−0.19–0.37]	0.525
Baseline eGFR	−0.16 [− 0.47–0.16]	0.317	0.00 [− 0.00–0.01]	0.695
Age	0.37 [− 0.59–1.33]	0.435	0.01 [0.00–0.03]	0.031
ACE inhibition	−2.63 [− 23.48–18.21]	0.798	− 0.08 [− 0.36–0.20]	0.574
Sex, female	−9.67 [− 29.06–9.71]	0.316	0.11 [− 0.15–0.37]	0.384

Estimated glomerular filtration rate [eGFR], urine protein creatinine ratio [uPCR], angiotensin converting enzyme [ACE], confidence interval [CI]

### Histological changes from baseline to repeat renal biopsy

After 6 months, 21/38 (55%) patients consented to and underwent a repeat renal biopsy. Interval changes in histology are recorded in Table 5. There was no statistically significant difference in histological progression for any feature between the 2 groups.

**Table 5:** Improvement in histological feature

	ART + C *N* = 12			ART alone *N* = 9			
Histological feature	Baseline Histology [*n* = 21]	Repeat biopsy [*n* = 12]	Repeat biopsies that improved (%)	Baseline histology [*n* = 17]	Repeat Histology [*n* = 9]	Repeat biopsies that improved (%)	*P* Value^a^
Lymphocytic infiltration			50.0%			55.6%	1.000
0	4.8%	0.0%		5.9%	22.2%		
1	8.6%	58.3%		41.2%	44.4%		
2	23.8%	33.3%		29.4%	33.3%		
3	28.6%	8.3%		11.8%	0.0%		
4	14.3%	0.0%		11.8%	0.0%		
Plasma cell infiltration			75%			44.4%	0.203
0	4.8%	8.3%		5.9%	11.1%		
1	28.6%	25.0%		29.4%	66.7%		
2	9.5%	41.7%		29.4%	11.1%		
3	19.0%	25.0%		5.9%	11.1%		
4	38.1%	0.0%		29.4%	0.0%		
Interstitial fibrosis			75.0%			44.4%	0.203
0	0.0%	0.0%		11.8%	33.3%		
1	14.3%	50.0%		35.3%	44.4%		
2	14.3%	41.7%		11.8%	22.2%		
3	23.8%	8.3%		29.4%	0.0%		
4	4.8%	0.0%		11.8%	0.0%		
Microcysts			41.8%			22.2%	0.642
0	23.8%	50.0%		41.2%	77.7%		
1	33.3%	50.0%		23.5%	11.1%		
2	33.3%	0.0%		23.5%	11.1%		
3	9.5%	0%		11.8%	0%		
4	0.0%	0%		0%	0%		

*ART + C* Antiretrovirals + corticosteroids, *ART Alone* Antiretrovirals alone, a = Fisher’s Exact Test to compare improvements in histology on repeat biopsy between groups

### Adverse events

**Fig. 4:**
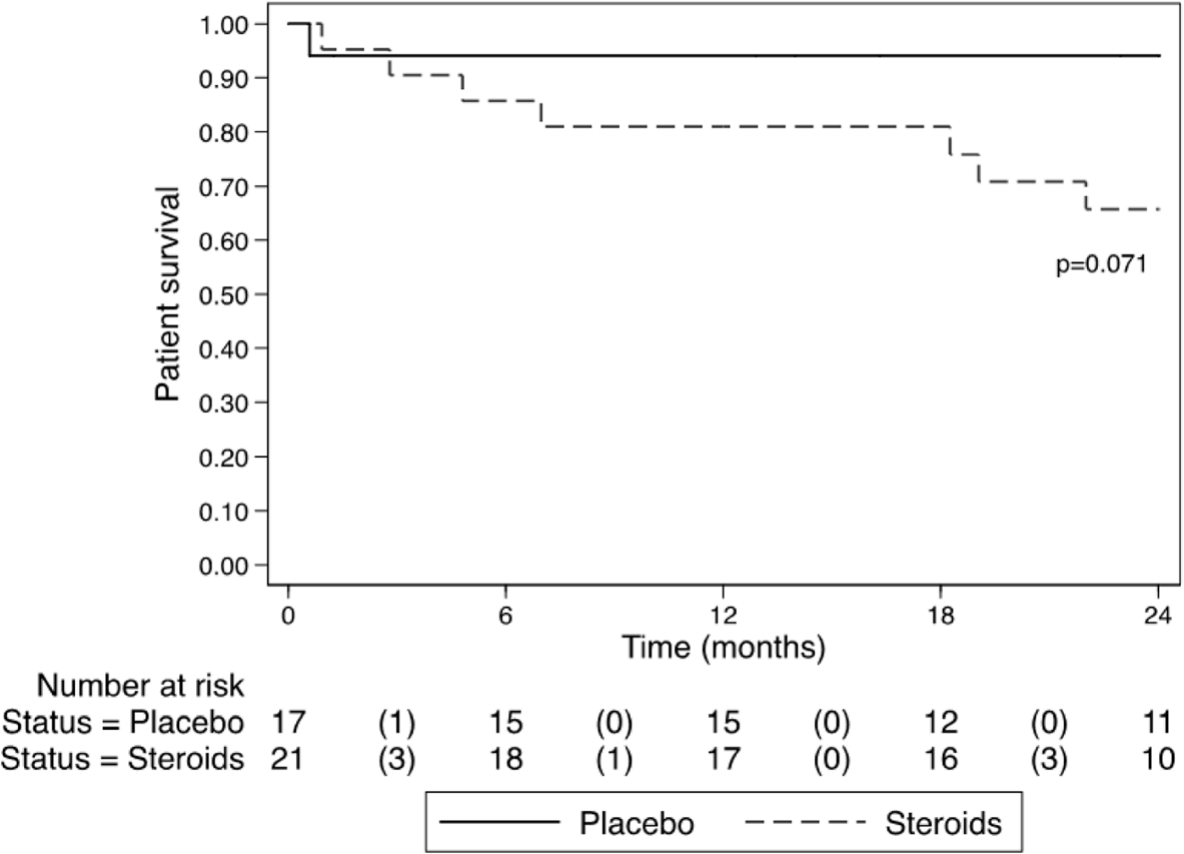
Kaplan Meier: Mortality [ART + C] vs [ART Alone]

There were 2 cases of herpes zoster that occurred between 10 and 14 days after commencing [ART + C]. Both cases were treated without sequelae. There were 8 deaths that occurred during the 24-month trial period, 1 from the group [ART Alone] and 7 from those treated with corticosteroids [ART+C] (Log rank *p* = 0.071) [Fig. 4: Kaplan Meier: Mortality [ART + C] vs [ART Alone]]. One death occurred in the first month in the group [ART Alone] as a result of TB. In the group [ART+C]: 1 patient died in the first month of the trial of unknown cause, 3 patients died of sepsis within the first 7 months of the trial and the other 3 patients died after completing corticosteroids. [See Additional file 3 Table S3].

## Discussion

Our study is the first open labelled prospective trial in Africa to assess the effect of corticosteroids on kidney function in patients with HIVAN treated at a single centre in Cape Town, South Africa. Important observations from this study include the following: (i) a significant increase in eGFR in patients treated with corticosteroids (without a significant reduction in proteinuria at last follow up) (ii) increased adverse events including risk of infections and all-cause mortality in the group treated with ART and adjuvant corticosteroids, and (iii) reduced interstitial inflammation seen on repeat biopsy in both arms without a significant difference between the groups.

Previous studies have demonstrated a positive effect of corticosteroids on kidney function in patients with biopsy proven HIVAN [22–24]. In a study of 20 patients with HIVAN on dual ART therapy with oral prednisone, Smith et al. reported a reduction of serum creatinine as well as a significant reduction of proteinuria [22]. However, relapses were reported when prednisone was tapered and six patients developed serious infections on prednisone, with 11 reported deaths [22]. In another study of 21 patients, 12 of which were treated with prednisone, Eustace et al reported an 80% reduction in risk of ESRD, but a higher risk of infections in the treatment group [23]. Only one retrospective study of 19 patients has assessed the use of corticosteroids in the triple therapy era of ART. The 5 patients who received prednisone experienced an increase in creatinine clearance of 5.57 ml/min/1.73 m^2^ per month (*p* = 0.003) [24].

The rational for the corticosteroid regimen used in our trial was based on the dosing schedule used to treat drug induced acute interstitial nephritis [AIN] as well as a regimen similar to that used by Smith et al. for steroid use in HIVAN [22, 26]. The tapering period was lengthened to a gradual taper over 6 months as the average length of time for HIV viral suppression after initiation of ART is 3–6 months [27–29]. The working hypothesis was that improving interstitial inflammation would improve renal function. Since HIV is the cause of interstitial inflammation in HIVAN it was important to allow enough time for the HIV to be controlled prior to cessation of the steroids.

This study demonstrates a positive improvement in eGFR in those receiving [ART + C] compared with those on [ART Alone] [Δ = 25mls/min (IQR: 15–51) vs 9 mls/min (IQR: 0–24), *p* = 0.008]. However, when the multivariable linear regression model was adjusted for baseline eGFR, although the change in eGFR over the study period was no longer significant the β coefficient [18.63] still demonstrated an improvement compared to ART alone. The lack of statistical significance is likely due to small numbers and the marked discrepancy of baseline eGFR between the two cohorts. Although proteinuria improved there was no statistical significant difference between the groups. On interval analysis, inflammation improved in both groups. Interstitial inflammation is a prominent histopathologic component of HIVAN [9, 11]. Inflammation may lead to interstitial fibrosis which is strongly correlated with poor renal prognosis in many forms of CKD including HIVAN [11, 30, 31]. .The lack of additional improvement of corticosteroids on proteinuria while improving eGFR may point to a benefit in dampening down the interstitial inflammatory response. The renal biopsy may have been performed too early and the sample size too small to see a significant response to those treated with corticosteroids.

There were 10 cases of TB identified prior to trial initiation. There were no further cases documented throughout the trial. Those with TB continued and completed their course of treatment. There were adverse events documented in the trial. There were 2 cases of herpes zoster treated without sequelae, 2 weeks after commencing ART and corticosteroids. There were deaths occurring in the early trial period in both groups with more deaths occurring in the corticosteroid arm. This could not be explained by differences in CD4 count or viral load suppression between the groups. [See Additional file 1 Table S1] This raises the question of a significant contributory effect of corticosteroids to sepsis in these patients.

However in contrast to our findings, a study conducted in South Africa using corticosteroids for the adjuvant treatment of tuberculous pericarditis used substantially higher doses of steroids [32]. This study had high numbers of HIV-infected patients and demonstrated a significant reduction in the incidence of constrictive pericarditis with no increased risk of mortality in those patients receiving corticosteroids. The trial did however demonstrate an increase in HIV associated cancers a finding not observed in our study [32]. The results of our trial suggest the need to reconsider the dose and duration of corticosteroid therapy. However, given that renal replacement therapy is rationed or limited in many regions of Africa the positive effect seen on kidney function needs to be balanced against the likelihood of sepsis and mortality.

There were several limitations of the study. Firstly, the sample size was small and the baseline eGFR was significantly lower in the corticosteroid arm. The randomization process used was via sealed envelope, although not ideal, was strictly adhered to. This highlights the need for a larger study defining hard endpoints of eGFR and proteinuria improvements with stratification into eGFR subgroups, to improve the validity of the results. Secondly patients and clinicians were not blinded to those who received corticosteroids, however the reason for this was additional opportunistic prophylaxis was required for this group. Thirdly at the start of this study there was no consensus on the histological definition of HIVAN. Fourthly, the length of time of follow up for the cohort was 24 months. For meaningful renal survival data, a longer follow up time period would be necessary. Lastly, pill counts were not used to assess compliance on corticosteroids.

## Conclusions

Despite the observed improvement in eGFR in patients treated with corticosteroids, our study cannot currently justify the use of corticosteroids at 1 mg/kg with tapering over 6 months to treat patients with biopsy proven HIVAN due to the increased mortality seen. Given the lack of effect on proteinuria it is postulated that the positive effect on eGFR may be having an impact on the interstitial component seen in HIVAN. Future longitudinal studies need to be directed to understanding those patients most likely to benefit from corticosteroid therapy while identifying those at higher risk for septic events. A lower corticosteroid dose, shorter duration of therapy and closer follow up may improve outcomes in this patient group.

## Additional files

Additional file 1: **Table S1.** CD4 count values and viral load non-suppression between baseline and 24 months. (DOCX 30 kb)

Additional file 2: **Table S2.** Baseline and last follow-up eGFR for all patients. (DOCX 32 kb)

Additional file 3: **Table S3.** Adverse Events. (DOCX 29 kb)

## Abbreviations

3TC: Lamivudine
ACEi: Angiotensin converting enzyme inhibition
ARB: Angiotensin receptor blockade tuberculosis (TB)
ART + C: Antiretroviral therapy plus corticosteroids
ART: Antiretroviral therapy
CD4: Cluster of differentiation [cells/mm^3^] cells per millimeter cubed
CKD: Chronic kidney disease
CKDepi: Kidney disease epidemiology collaboration
D4T: Stavudine
DBP: Diastolic blood pressure
EFV: Efavirenz
eGFR: Estimated glomerular filtration rate
ESRD: End-stage renal disease
g/dl: Grams per deciliter
H&E: Haematoxylin and eosin
HIV: Human immunodeficiency virus
HIVAN: HIV associated nephropathy
IQR: Interquartile range
KDIGO: Kidney disease improving global outcomes
Mg: Milligrams
Mmol/L: Millimoles per litre
PAS: Periodic acid-Schiff
SBP: SYSTOLIC blood pressure
TB: Tuberculosis
uPCR: Urine protein creatinine ratio
μmol/L: Micromoles per litre
