# Supplementary material for: The effects of add-on corticosteroids on renal outcomes in patients with biopsy proven HIV associated nephropathy: a single centre study from South Africa
Source: BMC Nephrol. 2019 Feb 6;20:44. doi: 10.1186/s12882-019-1208-2 (PMC6366071; doi:10.1186/s12882-019-1208-2)
Supplement: Supplementary file 1 — Table S1. CD4 count values and viral load non-suppression between baseline and 24 months. (DOCX 30 kb) [file 12882_2019_1208_MOESM1_ESM.docx]

|  | | | | | |
| --- | --- | --- | --- | --- | --- |
|  | **[ART +C]** | | **[ART ALONE]** | | **P Value** |
| **CD4 [median]** cells/mm3 | | | | | |
|  | **(n)** | **Mdn(IQR)** | **(n)** | **Mdn(IQR)** |  |
| **Baseline** | 21 | 133 [36-309] | 17 | 173 [104-352] | 0.454 |
| **3-6 months** | 11 | 214 (82-234) | 5 | 399 (250-788) | 0.1002 |
| **6-12 months** | 12 | 314 (193-505) | 11 | 384 (325-462) | 0.295 |
| **12-18 months** | 6 | 248 (57-483) | 11 | 350 (277-428) | 0.315 |
| **18-24 months** | 6 | 325(260-402) | 5 | 387 (355-423) | 0.465 |
| **Viral load : (Not -suppressed)*** | | | | | |
|  | **(n)** | **n (%)** | **(n)** | **n(%)** |  |
| **Baseline** | 15 | 15/15 (100%) | 15 | 100% | 0.000 |
| **3-6 months** | 11 | 2/11 (18%) | 5 | 1/5(20%) | 0.705 |
| **6-12 months** | 11 | 1/11 (9%) | 11 | 0/11 (0%) | 0.5% |
| **12-18 months** | 5 | 0/5 (0%) | 11 | 0/11 (0%) | 0 |
| **18-24 months** | 8 | 0/8 (0%) | 6 | 1/6 (17%) | 0.429 |
| * Viral load “Not suppressed”: refers to % of patients with viral load >100 copies/ml, a= Fisher’s Exact test | | | | | |
