# Supplementary material for: The effects of add-on corticosteroids on renal outcomes in patients with biopsy proven HIV associated nephropathy: a single centre study from South Africa
Source: BMC Nephrol. 2019 Feb 6;20:44. doi: 10.1186/s12882-019-1208-2 (PMC6366071; doi:10.1186/s12882-019-1208-2)
Supplement: Supplementary file 2 — Table S2. Baseline and last follow-up eGFR for all patients. (DOCX 32 kb) [file 12882_2019_1208_MOESM2_ESM.docx]

| **ART + Corticosteroid** | | | | **ART Alone** | | | |
| --- | --- | --- | --- | --- | --- | --- | --- |
| **Study number** | **Baseline**  **eGFR** | **Last follow up eGFR** | **Comment** | **Study number** | **Baseline**  **eGFR** | **Last follow up eGFR** | **Comment** |
| 18 | 22 | 51 | Died: at 7 months | 1 | 44 | 60 | Last eGFR =16 months[LtFup] |
| 19 | 116 | 125 | 24 months | 2 | 70 | 58 | 24 months |
| 20 | 32 | 59 | 23 months | 3 | 92 | 141 | 24 months |
| 21 | 51 | 64 | 24 months | 4 | 111 | 120 | 24 months |
| 22 | 86 | 123 | 24 months | 5 | 29 | 15 | Died at 1 month [0.59 months] |
| 23 | 12 | 72 | 24 months | 6 | 39 | 26 | Last eGFR=14 month[LTFup] |
| 24 | 18 | 113 | 24 months | 7 | 127 | 146 | Last eGFR =13 month[LTFup] |
| 25 | 18 | 38 | 24 months | 8 | 26 | 55 | 24 months |
| 26 | 35 | 99 | 24 months | 9 | 46 | 71 | 23 months |
| 27 | 44 | 113 | 22 months | 10 | 102 | 111 | 24 months |
| 28 | 46 | 46 | 24 months | 11 | 43 | 51 | 24 months |
| 29 | 63 | 105 | Died at 22 months | 12 | 47 | 71 | 24 months |
| 30 | 17 | 38 | Died: at 3 months | 13 | 14 | 24 | 24 months |
| 31 | 35 | 58 | Died: at 5 months | 14 | 35 | 35 | 24 months |
| 32 | 15 | 16 | Died: at 1 month | 15 | 101 | 90 | 24 months |
| 33 | 32 | 63 | 24 months | 16 | 97 | 103 | Last eGFR =1.25month [LTFup] |
| 34 | 14 | 134 | 22 months | 17 | 83 | 89 | 24 months |
| 35 | 46 | 61 | Died: at 18 months |  |  |  |  |
| 36 | 15 | 17 | 24 months |  |  |  |  |
| 37 | 46 | 69 | Last eGFR =12 months [LTfup] |  |  |  |  |
| 38 | 35 | 49 | Died: at 19months |  |  |  |  |
| [LTFup] Lost to follow up; [eGFR] estimated Glomerular Filtration Rate | | | | | | | |
