# Supplementary material for: The effects of add-on corticosteroids on renal outcomes in patients with biopsy proven HIV associated nephropathy: a single centre study from South Africa
Source: BMC Nephrol. 2019 Feb 6;20:44. doi: 10.1186/s12882-019-1208-2 (PMC6366071; doi:10.1186/s12882-019-1208-2)
Supplement: Supplementary file 3 — Table S3. Adverse Events. (DOCX 29 kb) [file 12882_2019_1208_MOESM3_ESM.docx]

|  | **n** | **Details** | **Time frame** | **Outcome and causes of death** |
| --- | --- | --- | --- | --- |
|  |  |  |  | Outcomes |
| **Morbidity**:  [ART + C] | 2 | Herpes Zoster | 10-14 days after corticosteroids commenced | Successful treatment |
| **Mortality** | 8 |  |  | Causes of Death |
| ART Alone | 1 |  | Occurred within 1 month of starting antiretroviral therapy | From Tuberculosis |
| ART + C | 7 | 1 patient | 1 month from start of trial | Unknown cause |
|  |  | 3 patients | First 7 months | Overwhelming sepsis |
|  |  | 3 patients | After completing steroids [>18 Months] | 1 patient had disseminated TB  2 patients had overwhelming sepsis |
